# Complex History of Codiversification and Host Switching of a Newfound Soricid-Borne Orthohantavirus in North America

**DOI:** 10.3390/v11070637

**Published:** 2019-07-11

**Authors:** Schuyler W. Liphardt, Hae Ji Kang, Laurie J. Dizney, Luis A. Ruedas, Joseph A. Cook, Richard Yanagihara

**Affiliations:** 1 Department of Biology and Museum of Southwestern Biology, University of New Mexico, Albuquerque, NM 87131, USA; 2 Department of Pediatrics, John A. Burns School of Medicine, University of Hawaii at Manoa, Honolulu, HI 96813, USA; 3 Department of Biology, University of Portland, Portland, OR 97203, USA; 4 Department of Biology and Museum of Vertebrate Biology, Portland State University, Portland, OR 97207-0751, USA

**Keywords:** *hantaviridae*, orthohantavirus, hantavirus, shrew, viral evolution, codiversification

## Abstract

Orthohantaviruses are tightly linked to the ecology and evolutionary history of their mammalian hosts. We hypothesized that in regions with dramatic climate shifts throughout the Quaternary, orthohantavirus diversity and evolution are shaped by dynamic host responses to environmental change through processes such as host isolation, host switching, and reassortment. Jemez Springs virus (JMSV), an orthohantavirus harbored by the dusky shrew (*Sorex monticola*) and five close relatives distributed widely in western North America, was used to test this hypothesis. Total RNAs, extracted from liver or lung tissue from 164 shrews collected from western North America during 1983–2007, were analyzed for orthohantavirus RNA by reverse transcription polymerase chain reaction (RT-PCR). Phylogenies inferred from the L-, M-, and S-segment sequences of 30 JMSV strains were compared with host mitochondrial cytochrome *b*. Viral clades largely corresponded to host clades, which were primarily structured by geography and were consistent with hypothesized post-glacial expansion. Despite an overall congruence between host and viral gene phylogenies at deeper scales, phylogenetic signals were recovered that also suggested a complex pattern of host switching and at least one reassortment event in the evolutionary history of JMSV. A fundamental understanding of how orthohantaviruses respond to periods of host population expansion, contraction, and secondary host contact is the key to establishing a framework for both more comprehensive understanding of orthohantavirus evolutionary dynamics and broader insights into host–pathogen systems.

## 1. Introduction

Comparative phylogeographic study of parasites and their hosts can provide valuable insights into evolutionary history and processes of diversification for both host and parasite. These analyses also provide a predictive framework for emerging disease under scenarios of changing environmental conditions [1,2,3]. The classic model of strict host–parasite codiversification as the leading process driving parasite evolution has been thrown into question over the past several decades with studies suggesting that host switching is not only more prevalent than originally thought, but that it may be the most common pattern throughout parasite evolutionary history [4,5].

Viruses are hypothesized to follow processes of diversification in a manner similar to organisms more traditionally considered to fulfil the ecological role of parasites, such as helminths and bacteria. Members of the recently reclassified genus *Orthohantavirus* (order *Bunyavirales*, family *Hantaviridae*) [6] can cause severe disease in humans. These viruses have a complex history of both codiversification with their mammal hosts [7] and host switching across mammalian species [8,9]. Viral reassortment—the exchange of viral material between divergent strains [10,11,12]—is another process that can rapidly lead to novel variants (e.g., 2009 human influenza A H1N1 virus [13]). Reassortment may be frequent in orthohantaviruses; however, research to date has mainly focused on reassortment within orthohantavirus strains hosted by single species [10].

Jemez Springs virus (JMSV), originally discovered in the dusky shrew (*Sorex monticola*) in New Mexico [14], has subsequently been detected in other closely related members of the *Sorex vagrans* complex. The broad distribution of JMSV across western North America provides an ideal system to begin to examine the prevalence of viral codiversification, host switching, and reassortment within a mammalian species complex.

*Sorex monticola* (order Eulipotyphla, family Soricidae) is endemic to North American montane forests in the western United States, Canada, and Mexico, and is a member of the *Sorex vagrans* complex [15]. Demboski and Cook [16] suggested that rapid diversification early in the history of these mammals was stimulated by repeated population isolation and expansion events due to cyclical glaciation events in the Late Quaternary and the Last Glacial Maximum. This pattern is reflected in the geographic structure of clades elucidated by phylogenic analyses of *S. monticola* and close relatives in western North America [16], including the American water shrews, *Sorex palustris* and *Sorex navigator* [17], of which *S. palustris* also hosts JMSV.

Seewis virus in Finland also shows a strong signal of post-glacial expansion and codiversification of the virus with its host, the common shrew, *Sorex araneus* [18]. Phylogeographic studies of Seewis virus in Siberia suggest a similar pattern of codiversification [19], but with instances of host switching followed by localized variation in closely related species [20,21,22]. Similar patterns are found in Puumala virus with preliminary evidence for post-glacial colonization of Finland coinciding with expansion of the mammalian host, the bank vole (*Myodes glareolus*) [23]. However, subsequent studies that were more geographically extensive showed little evidence supporting strict codiversification of Puumala virus and the bank vole [24].

Phylogeographic study has helped to uncover the complex forces and interactions responsible for the maintenance, transmission, and diversification of viruses, especially those capable of causing disease in humans [25]. Here we examine a species complex broadly distributed throughout western North America to help bridge the gap between deep-time phylogenetic analyses of viral diversification across major mammalian clades and studies that examine relatively recent viral evolution within a single host species. We extend our understanding of processes and patterns of virus evolution and ecology by taking a comparative phylogeographic approach to gain insights into the evolutionary history of JMSV in *S. monticola* and its close relatives.

## 2. Materials and Methods

### 2.1. Specimens

We studied liver or lung tissues from 69 dusky shrews, 10 American water shrews, 17 vagrant shrews, 9 Trowbridge shrews (*Sorex trowbridgii*), and 1 prairie shrew (*Sorex haydeni*), archived in the Museum of Southwestern Biology at the University of New Mexico in Albuquerque (Table 1). Also tested were lung tissues of 28 vagrant shrews, 26 Trowbridge shrews, 3 Pacific water shrews (*Sorex bendirii*), and 1 Baird’s shrew (*Sorex bairdi*), captured in Multnomah and Washington counties, near Portland, Oregon, between April 2003 and October 2005, and archived at the Museum of Natural History, at the Portland State University (Table 1), as part of an epizootic study of Sin Nombre virus infection in deer mice (*Peromyscus maniculatus*) [26]. All tissue samples were frozen at –70 °C until tested by RT-PCR.

### 2.2. RNA Extraction, cDNA Synthesis, and RT-PCR Amplification

Total RNA was extracted from 20–50 mg of each tissue using the PureLink Micro-to-Midi total RNA purification kit (Invitrogen, San Diego, CA, USA). cDNA was prepared using the SuperScript III First-Strand Synthesis System (Invitrogen) and oligonucleotide primer (5’-TAGTAGTAGACTCC-3’) designed from the conserved 3’-end of the S, M, and L segments of orthohantaviruses.

Gene amplification was carried out in 20-μL reaction mixtures containing 250 μM dNTP, 2 mM MgCl_2_, 1 U of AmpliTaq polymerase (Roche, Basel, Switzerland), and 0.25 μM of oligonucleotide primers, designed from highly conserved regions of previously identified soricid-borne orthohantaviruses. A listing of the oligonucleotide primers used to amplify the S-, M-, and L-genomic segments is provided in Table S1. Initial denaturation was followed by touchdown cycling (two-degree step-down annealing from 48 °C to 38 °C for 40 sec) and elongation at 72 °C for 1 min, then 32 cycles of denaturation at 94 °C for 40 sec, annealing at 42 °C for 40 sec, and elongation at 72 °C for 1 min, in a GeneAmp PCR 9700 thermal cycler (Perkin-Elmer, Waltham, MA, USA). Amplified products were separated by electrophoresis on 1.5% agarose gels and purified using the QIAQuick Gel Extraction Kit (Qiagen, Hilden, Germany). DNA was sequenced directly using an ABI Prism 377XL Genetic Analyzer (Applied Biosystems Inc., Foster City, CA, USA).

### 2.3. Sequence Dataset

Virus sequences, either generated in this study or downloaded from GenBank [27] of all currently known strains of JMSV and their respective hosts spanning the range of JMSV, as well as outgroups for rooting of the trees, were studied (Figure S1A). The final dataset, with outgroups, was composed of partial coding regions for the L (*n* = 31), M (*n* = 10), and S (*n* = 29) segments of orthohantaviruses. In addition, mitochondrial cytochrome *b* (cyt *b*) gene sequences, generated for each mammalian host across the range of JMSV variation, were included to independently examine host phylogenetic relationships (Figure S1B). GenBank accession numbers for all sequences used in this study are in Table S2.

### 2.4. Phylogenetic Analysis

Phylogenetic trees were generated from alignments of each individual genomic segment (S, M, and L) and Ash River virus from *Sorex cinereus* [14] as an outgroup using Muscle version 8.1.9 [28], as implemented in Geneious 8.1 [29]. To detect instances of intergenic rearrangement and assure we were analyzing homologous genomic regions, these independent alignments were tested for recombination using the Phi [30], NSS [31], and Max χ^2^ [32] tests as implemented in PhiPack [33]. Both a maximum likelihood approach using RAxML version 8.2.12 [34], and Bayesian probability implemented in MrBayes version 3.2.5 [35], were employed for tree inference. A general time reversible (GTR) model of nucleotide evolution with gamma-distributed rate heterogeneity and invariable sites (RAxML option GTRGAMMAI) was determined to be the best fit model for this dataset by JModelTest [36]. RAxML generated 1000 bootstrap replicates to determine the best-fit maximum likelihood tree and associated nodal support. MrBayes was run for 10 million generations using the priors from JModelTest by sampling trees every 1000 generations. After the first 25% of trees were discarded as a recommended burn-in, the remaining trees were used to calculate a 50% majority rule consensus tree. Sequence alignments for all segments, as well as tree files in newick format, are available in supplementary materials.

### 2.5. Tanglegrams, Diversity Analyses, Codiversification Tests, and Reconciliation

Tajima’s D statistic for both virus and host alignments were computed in R [37] with the package Pegas [38]. Negative values for Tajima’s D can be indicative either of population expansion or of a selective sweep [39]. Individual pairwise distances and between group mean distances were calculated in Mega7 [40] with a Kimura 2-parameter model and 500 bootstrap replicates. To test codivergence versus host switching, several metrics that measure the extent of similarity between phylogenetic trees were employed. The nPH85 metric [5] is a normalized version of the Robinson Foulds tree topology distance that incorporates branch lengths and returns a value between 0 and 1, with 0 indicative of strict codiversification between identical tree topologies or 1 indicative of cross species transmission with completely incongruent tree topologies, and was calculated in the R package NELSI [41]. Trip [42] and TripL [43] measure the number of shared triplets between two trees, with TripL incorporating branch lengths and returning the proportion of triplets not shared between two trees, and was calculated in the R package Kaphi [44]. In this metric, 0 indicates identical trees and 1 indicates no shared triplets. Phylogenetic reconciliation was performed for the L and S segment with host cyt *b* phylogeny in Jane 4.01 [45] with equal costs of 1 assigned to all possible events. Finally, to visualize tree discordance, tanglegrams were generated for each tree comparison in R with the package phytools [46].

## 3. Results

### 3.1. Phylogenetic Analysis

**Figure 1:**
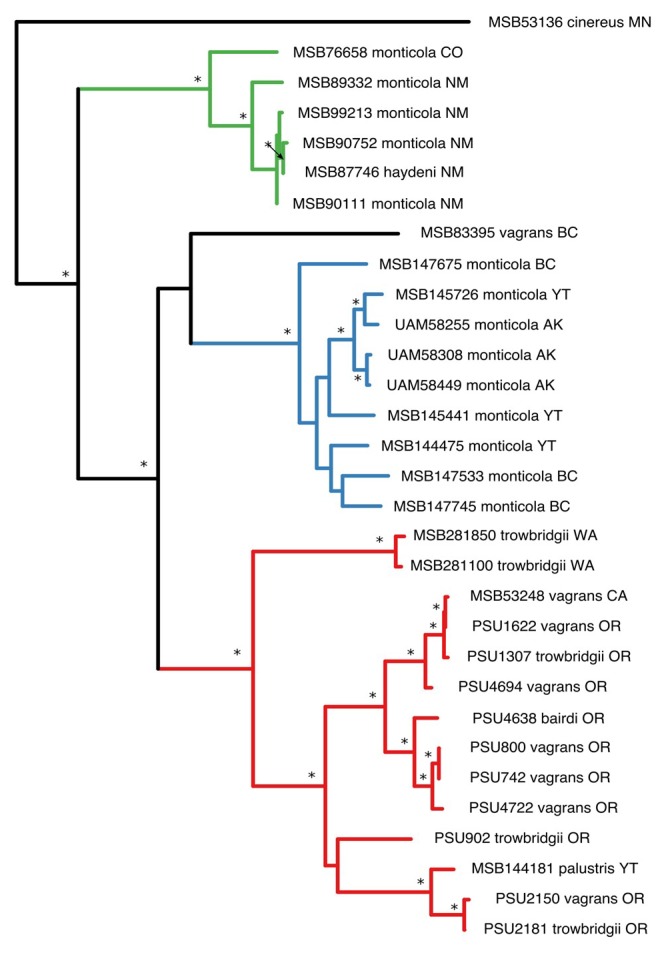
Maximum likelihood phylogeny of Jemez Springs virus L segment inferred using RAxML and rooted at the midpoint. Nodes with bootstrap support values greater than 70% are indicated with an asterisk. Color-coded branches correspond to geographic clades: Green = Southern Continental; blue = Northern Continental; red = Pacific Coastal. State abbreviations: AK, Alaska; BC, British Columbia; CA, California; MN, Minnesota; NM, New Mexico; OR, Oregon; WA, Washington; YT, Yukon Territory. GenBank accession numbers for the L-segment sequences are provided in Table S2.

**Figure 2:**
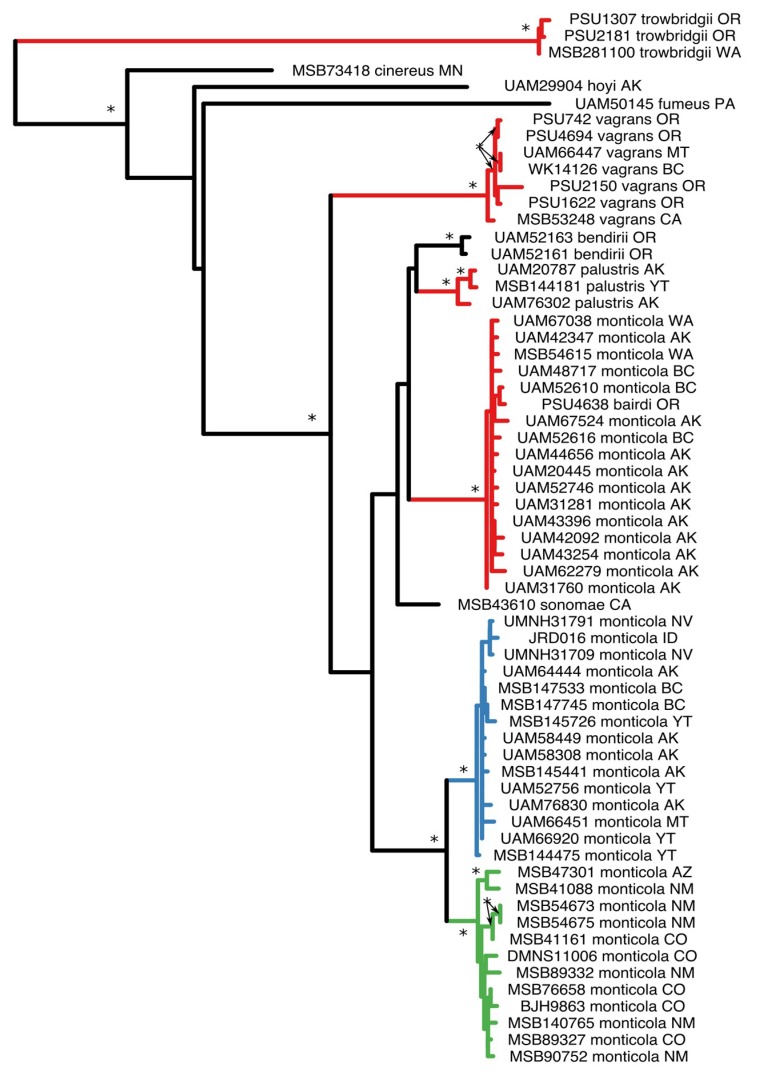
Maximum likelihood phylogeny of the *Sorex vagrans* complex cytochrome *b* inferred using RAxML and rooted at the midpoint. Bootstrap support values greater than 70% are indicated with an asterisk. Color-coded branches correspond to geographic clades of the virus: Green = Southern Continental; blue = Northern Continental; red = Pacific Coastal. State abbreviations: AK, Alaska; AZ, Arizona; BC, British Columbia; CA, California; CO, Colorado; ID, Idaho; MN, Minnesota; MT, Montana; NM, New Mexico; NV, Nevada; OR, Oregon; PA, Pennsylvania; WA, Washington; YT, Yukon Territory. GenBank accession numbers for the shrew cytochrome *b* sequences are available in Table S2.

Significant values for recombination analyses differed among segment and test used but identified no regions of intragenic rearrangement in any segment analyzed. The JMSV strains showed a pattern of diversification with defined clades that closely mapped to geographic regions similar to that seen in the phylogeographic structure of the hosts, with the exception of the virus recovered from *S. palustris*, which was previously identified as Fox Creek virus ([47]; Figure 1). Geographic regions correspond to Northern Continental (NC) and Southern Continental (SC) clades composed of viruses recovered strictly from *S. monticola*. The NC clade contained JMSV strains from British Columbia and Yukon Territory, as well as Alaska, while the SC clade was found in New Mexico and Colorado. A third clade was recovered from the Pacific coast (PC) which, in comparison to the NC and SC clades, was hosted by several species of the *S. vagrans* complex, including *S. vagrans, S. trowbridgii, S. bairdi,* and *S. palustris*. Phylogenies inferred using the L and S segment differed in regard to the placement of JMSV strains recovered from *S*. *vagrans* in Washington (Figure S3). For host cyt *b*, *S. trowbridgii* and *S. vagrans* were supported as monophyletic; however, *S. monticola* was shown to be paraphyletic, with a coastally distributed clade, including the sole *S. bairdi* sample, that is sister to *S. palustris*, and a larger continental clade that is composed of northern and southern subclades containing viruses from the NC and SC clades, respectively (Figure 2).

### 3.2. Population Demographics

Tajima’s D statistic for all three viral genomic segments, as well as host cyt *b*, were −3.8, −4.5, −3.8, and −3.1 for the S, M, L segments and host cyt *b*, respectively, with all values being highly significant (*P* < 0.01). Overall mean diversity, as computed in MEGA using a Kimura 2-parameter model, was 0.256. This relatively high level of nucleotide diversity, common in RNA viruses with high mutation rates, is driven mainly by the PC clade and between group divergence. Within clade distances were highest in the PC clade at 0.196 followed by the NC clade at 0.125. The SC clade had the lowest diversity at 0.053; however, this relatively low value could be an artifact of smaller sample size.

### 3.3. Cophylogeny Tanglegrams

**Figure 3:**
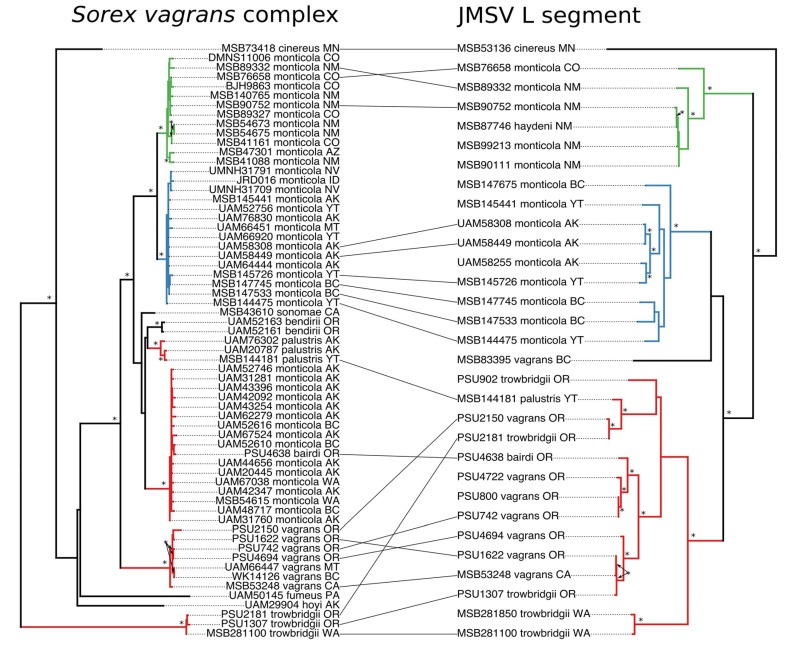
Tanglegram for *Sorex vagrans* complex on the left, and Jemez Springs virus L segment on the right. Host individuals and their associated virus strains are illustrated with a black line. Color-coded branches correspond to geographic clades of the virus: Green = Southern Continental; blue = Northern Continental; red = Pacific Coastal. State abbreviations are as shown in Figure 2. Bootstrap support values greater than 70% are indicated with an asterisk.

Cophylogeny of the L segment and host tree reflected a pattern of codiversification within continental *S. monticola*, but a complex pattern of host switching across multiple species within the PC clade. Host phylogeny based on cyt *b* placed the NC and SC clades as sister lineages forming a larger continental *S. monticola* group that matched the pattern of diversification seen in the L segment phylogeny (Figure 3). Included in the JMSV L segment PC clade were viruses harbored by *S. vagrans*, *S. trowbridgii*, and *S. bairdi* that corresponded to taxa spread across the host phylogeny. Of note was the placement of *S. bairdi* within the coastal *S. monticola* clade, which represented the only sample of JMSV from that clade. Additionally, JMSV recovered from *S. trowbridgii* was distributed across the JMSV PC clade, yet *S. trowbridgii* was only distantly related to the *S. vagrans* complex. *Sorex cinereus*, the host of Ash River virus, which was used as the outgroup to JMSV in this study, was more closely related to the *S. vagrans* complex than *S. trowbridgii*. Cophylogenies based on the M segment (Figure S2) and S segment (Figure S3) of JMSV showed similar patterns to those recovered with the L segment. However, phylogeny reconstruction based on a single gene for the hosts, in this case cyt *b*, can be misleading and should be further tested with additional, independent loci for this complex of shrew species.

In our study, all segments of JMSV supported monophyletic NC and SC clades that mapped to divergent, reciprocally monophyletic *S. monticola* clades in the host phylogeny and corresponded to the same geographic regions. While the shallow topology (i.e., branching near the terminal tips) seen within JMSV and the NC and SC clades of *S. monticola* differed and do not map one-to-one, the geographic clades were reciprocally monophyletic and generally supported a pattern of host–parasite codiversification within the continental group.

In contrast, the PC clade of JMSV showed a complex pattern of host switching among sympatric, yet often deeply divergent species of shrews along the Pacific coast. While the PC clade is monophyletic in all segments of JMSV, this clade is distributed across four separate host species, hence indicative of widespread host switching. Of note among the JMSV PC clade was the placement of a virus recovered from *S. palustris* (MSB144181) that was well supported within the PC clade. The host specimen was, however, collected in the Yukon Territory, well within the range of the NC clade of both JMSV and *S. monticola*. This pattern of host switching was mirrored by the JMSV strains hosted by *S. trowbridgii* and *S. vagrans* that were spread across the PC clade and did not form a single species-specific clade. Virus–virus cophylogenies based on the L and S segments largely mirrored each other with the exception of the placement of two JMSV strains recovered from *S. trowbridgii* from Washington, which likely was an artifact of sequence coverage for those two segments (Figure S6). A single virus recovered from a *S. vagrans* (MSB83395) specimen from Vancouver Island, British Columbia, was not supported as being a member of any of the three geographic clades within JMSV (Figure 3 and Figure S4). It is important to note, however, that these analyses are based on tree topologies that are not fully resolved, resulting in uncertainty regarding sister relationships within both the host and viral trees, which can impact inferences of codiversification and host switching within this system. More sampling is necessary to resolve tree topologies and fully elucidate the evolutionary history of both the shrew hosts and JMSV.

### 3.4. Codivergence and Phylogenetic Reconciliation

We compared metrics that test for codivergence as they fundamentally differ in their methods of calculating tree similarity. The nPH85 statistic was similar for both S and L segments: Their relatively high values, 0.80 and 0.76, respectively, indicated host switching rather than codiversification as the leading pattern of diversification within JMSV. Codiversification previously has been reported as the more prevalent coevolutionary pattern within *Bunyavirales* [5]. That pattern, however, is not necessarily reflected in the TripL and Trip metrics. For the L segment, the TripL and Trip metrics were 0.41 and 0.44, respectively, indicating that the L segment and host phylogenies shared roughly 60% of triplets. This result differs from the TripL and Trip metrics for the S segment at 0.23 and 0.19, respectively, relating to roughly 80% of shared triplets between the S segment and host phylogenies.

**Figure 4:**
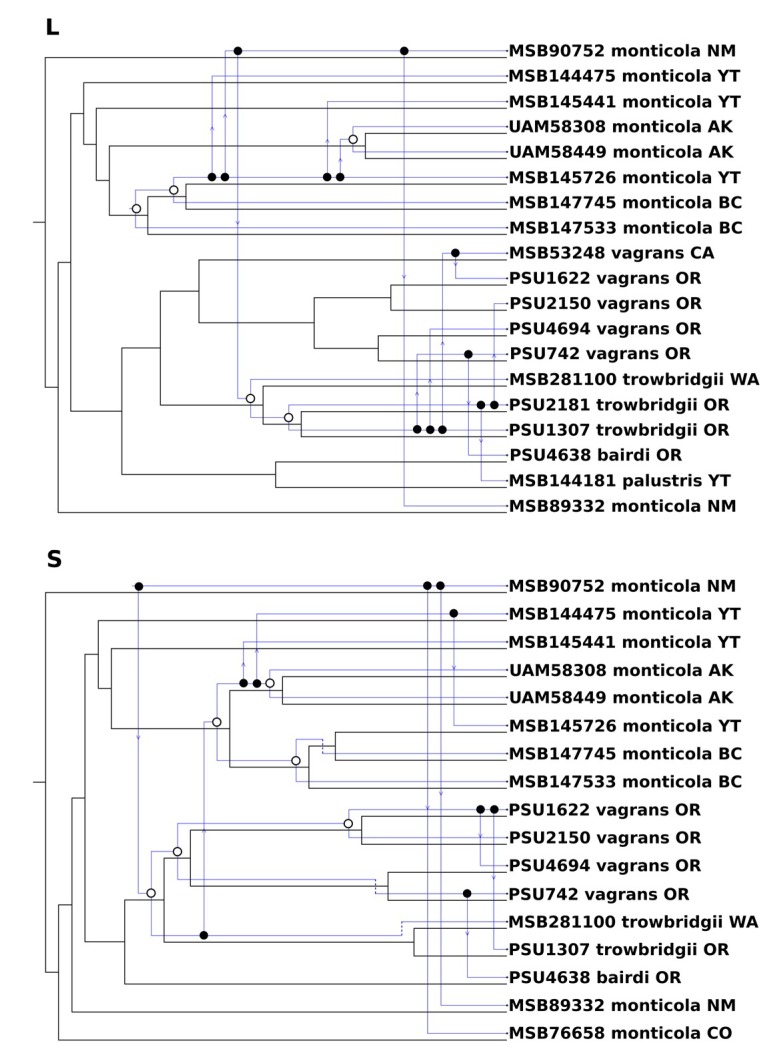
Phylogenetic reconciliation for the Jemez Springs virus L and S in blue and the *Sorex vagrans* complex phylogeny in black. Open circles represent codivergence events, solid circles followed by an arrow indicating host-switching events and direction, and dashed lines represent virus extinction events. State abbreviations are as shown in Figure 1.

In contrast to the metrics of codivergence between the L or S segment with the host phylogeny, the metrics comparing the L segment to the S segment in some cases show greater similarity between virus and host than between segments of JMSV. While the nPH85 metric for the S and L comparison is less than that for either comparison with the host, it is still relatively elevated at 0.5 indicating that the two segments share roughly half of their internal structure to each other. Also striking is the TripL and Trip scores for the comparison between segments at 0.46 and 0.49, respectively, also indicating that in addition to the variation on internal tree structure, the S and L segment only share about half of their triplets, less than the S segment shares with the host phylogeny. Differences in evolutionary history between the S and L segments of JMSV are reflected by phylogenetic reconciliation for each segment with the host phylogeny (Figure 4). These segments suggest that a distinctive set of host switching, codivergence, and local extinction events, are necessary to reconcile the virus segment phylogeny with the host phylogeny. This pattern of independence between segments is consistent with a history of reassortment among the L and S segments of JMSV.

## 4. Discussion

### 4.1. Codiversification Processes

Comparative phylogeography of viruses and their associated mammal reservoir hosts can shed light on the processes driving patterns of coevolutionary diversification [2,48] by revealing the role of historical events that shaped contemporary diversity. Contact between divergent hosts may facilitate transmission of viruses to novel hosts, or reassortment of divergent viral components. Comparative phylogeographic studies provide the spatial and temporal foundation necessary for understanding viral evolution, transmission, and disease emergence; these are essential tools for researchers and public health agencies to proactively approach disease emergence and mitigation. In this study, we addressed the evolutionary history that shaped modern diversity within JMSV and associated mammals hosting this virus.

Diversification within JMSV largely reflects the recent biogeographic history of the shrew host species. The phylogeny inferred from cyt *b* for the *S. vagrans* complex supports previously reported species designations and relationships [16]. Demboski and Cook [16] recovered substantial geographic structure within *S. monticola*, identifying distinct clades distributed in northern and southern continental North America, and a third distributed along the Pacific coast. Representative clades in JMSV match the NC and SC clades, respectively, while the PC clade is comprised of viruses recovered from several other species in the *S. vagrans* complex, including *S. bairdi* which falls within the Pacific coastal *S. monticola* clade. That pattern ostensibly parallels two suggested evolutionary diversification events within the *S. vagrans* species complex [16]. To date, the absence of JMSV in *S. monticola* specimens representative of the PC clade (Oregon and Washington) may reflect either true absence or simply low sampling coverage. The validity of *S. bairdi* as a species separate from coastal *S. monticola* is questionable and points to other poorly defined species limits in this shrew complex that complicate our assessment [16]. Expanded shrew sampling and viral screening that aims to refine the geographic extent of host limits and viral diversity in western North America are necessary.

**Figure 5:**
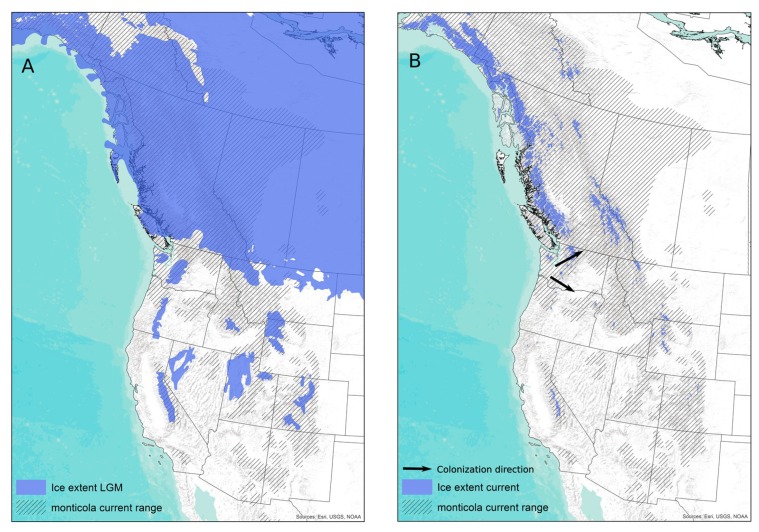
(**A**) Extent of ice sheets across western North America during the Last Glacial Maximum and the modern distribution of *Sorex monticola*. (**B**) Current glacial extent of western North America according to the Global Land Ice Measurements from Space (GLIMS) [52], modern distribution of *Sorex monticola*, and the hypothesized direction of post-glacial expansion of *S. monticola* to the North and South.

The hypothesized initial divergence event within the *S. vagrans* complex occurred in the Pacific Northwest coast resulting in current inter-species diversity seen within the complex. Subsequent post-glacial expansion followed the Last Glacial Maximum and produced the currently recognized geographic structure within montane shrews (e.g., NC and SC clades) and their close relatives (e.g., *Sorex palustris* [17]) (Figure 5). With JMSV largely mirroring the host pattern, codiversification between JMSV and its shrew hosts appears likely within the NC and SC clades. JMSV emergence tentatively can be dated to the initial diversification of the *S. vagrans* complex ca. 2 MYA [16], with subsequent, possibly multiple independent, post-glacial expansion events within *S. monticola*, and likely *S. palustris*, during the mid to late Quaternary. Whether the virus recovered from *S. palustris* within the PC clade is the result of a recent host-switching event, is unclear, and more sampling is necessary to refine our understanding of the evolutionary history of JMSV within *S. palustris*. The single virus recovered from a *S. vagrans* on Vancouver Island does not align within a defined mainland clade in the S segment and exists on a long branch in the L segment. This finding raises the question of whether there is an endemic insular clade of JMSV similar to that identified for insular *S. monticola* [49]. Highly negative Tajima’s D values for all three viral segments, coupled with observed patterns of sequence divergence centered in the hypothesized source population of the PC clade, strengthens the hypothesis of post-glacial expansion for JMSV (Figure 5B). While the timing of orthohantavirus diversification remains elusive [7,50,51], this shared pattern of diversification, both spatially and temporally, between JMSV and its mammalian hosts is consistent with a cophylogeographic history that is much deeper than several thousand years.

While phylogenies for JMSV and its host species largely mirror each other in deeper phylogenetic structure, codiversification is only partially responsible for contemporary diversity. Elevated codivergence metrics calculated between trees suggest host switching also plays an important role. Geoghegan and colleagues [5] applied the nPH85 metric to family level phylogenies for several RNA and DNA viruses. Our results for this metric largely match the general pattern seen for other RNA viruses [5]. When comparing topologies between the host phylogeny and either the S or L segment, we calculated an nPH85 value of 0.8 and 0.76, indicating scant codivergence, which is consistent with the values seen at the family level. Furthermore, when calculated for the comparison between the L and S segments, we obtained a value of 0.5, indicating a mix of codivergence and host switching between each segment. This is in contrast with the tanglegram for the L and S segment comparison (Figure S5) which indicated the phylogenies largely mirrored each other. However, the level of similarity between tree topology and groupings of terminal taxa do not necessarily tell the same story as indicated by drastically different TripL and Trip values compared to nPH85. Internal branching structure is driving the difference in codivergence metrics calculated between phylogenies inferred from the L and S segments. However, our sampling of the L segment is more complete in terms of number of specimens and coverage of the segment. This fact, coupled with our phylogenetic reconciliation analysis, suggests that in addition to a complex history of post-glacial codivergence and local host switching between JMSV and the *S. vagrans* complex as a whole, there is additional complexity due to independent evolutionary histories associated with distinct viral segments. Increased sampling and full-length genomic sequencing would help test whether incongruity between the S and L segments, both tree topology and similarity metrics, reflects deeper phylogenetic patterns or is merely an artifact of sampling and sequencing bias.

### 4.2. Viral Reassortment

Viral reassortment is possible for viruses with segmented genomes and can be a catalyst for driving diversification and pathogenesis. The 2009 influenza outbreak is a prime example of reassortment among multiple divergent strains resulting in a pandemic [13]. Reassortment produces unique combinations of viral segments that have the potential to influence pathogenicity due to presentation of novel virions to an immunologically naïve population. Co-circulation of divergent viruses within a single cell is hypothetically necessary for reassortment and calls for a more detailed understanding of the range of ecological circumstances that can lead to viral switching between hosts if we hope to predict disease emergence [2].

Historically, reassortment events within orthohantaviruses were thought to be relatively rare compared to other members of *Bunyavirales* due to method of transmission (i.e., direct host–host contact versus arthropod transmission, respectively) [12]. However, an increasing number of studies of orthohantaviruses have shown instances of reassortment [53,54,55,56], both ancient and modern. The extent of reassortment and its contribution to modern-day orthohantavirus diversity is a new avenue of research and there is still much to learn.

Implicit in orthohantavirus reassortment events is the necessity for contact between hosts for transmission of divergent viruses, or what are called spillover events. For instance, there is evidence in Finland for two distinct strains of Puumala virus co-circulating with active and ongoing reassortment resulting from contact, both modern and historic, between divergent populations of the host species, *M. glareolus* [54]. A more contemporary example of co-circulation was reported in Belgium with two deeply divergent strains of *Hantaviridae* co-circulating in the European mole, *Talpa europaea* [57]. That system is unique in that multiple strains of virus have been recovered from a single individual host sample, potentially representing a prime situation for reassortment. Such scenarios, where reassortment and host-switching events can occur, highlight that studies that expand our knowledge of both geographic and host range are crucial to gaining a better understanding of the role of host ecology and evolutionary history in viral spread, emergence, and pathogenesis.

Incongruence in tree topologies between the L and S segments likely reflects host-switching and reassortment in the evolutionary history of JMSV. The large geographic distance between the NC and SC clades (Canada/Alaska and New Mexico/Colorado, respectively) containing the possible reassortant strain suggests that this event likely predates the post-glacial expansion of *S. monticola*. However, the lack of sampling spanning the distance between the NC and SC clades, minimal sampling for the M segment to date, and only a single instance of reassortment, precludes full elucidation of its role in the evolutionary history of JMSV. To attain a more complete history of JMSV, and shrew-borne orthohantaviruses in general, a comprehensive sequence dataset of all three genomic segments is needed, spanning the breadth of host diversity. Klempa showed that reassortment among orthohantaviruses is more prevalent than originally believed [10], however, the study of orthohantavirus reassortment is relatively under-explored and the extent of this process in orthohantavirus diversification remains largely unknown.

Shi and colleagues [58] provided a better understanding of the deeper phylogenetic and evolutionary history responsible for the current diversity in vertebrate viruses at the order and family level, revealing that an overall trend of codivergence is coupled with a complex history of host switching between distantly related taxa. Bennett and co-workers [47] showed that these trends of host switching and codivergence hold true within a single virus genus, *Orthohantavirus*, in their exploration of the relationships and phylogeographic history of several strains of orthohantavirus. In addition, Torres-Pérez and colleagues [7] examined the phylogeographic history and patterns of divergence within a single strain and host, Andes virus hosted by the South American rodent, *Oligoryzomys longicaudatus*. Their results revealed that while there was overall similarity in spatial structure between virus and host phylogenies, the timing of diversification was incongruent. That research highlights the difficulty of accurately dating the evolution of hantaviruses, with estimates ranging broadly from several thousand to several million years before present [50,59,60,61]. Our study of a single orthohantavirus that is shared among multiple soricid host species begins to explore the role of host history across a temporal scale that spans across population-level processes to much deeper evolutionary events.

JMSV does not show a history of strict codiversification, but rather multiple host-switching events at both broad and fine geographic scales, and at least one instance of reassortment of divergent strains. More sampling is necessary to elucidate the evolutionary history of JMSV within *S. palustris, S. bairdi*, and other close relatives of this shrew complex, and would help resolve uncertainty of tree topology which limits our ability to fully understand this system. Nonetheless, it is evident that JMSV has a complex and relatively deep evolutionary history in North America. That history remains complicated by uncertainty of host taxonomy, such as polyphyletic assemblages in both *S. monticola* and *S. palustris* [16,17]. Such uncertainty illuminates the necessity for a solid understanding of host relationships and history when discerning the evolutionary history of viruses and parasites in general.

## Figures and Tables

**Table 1:** RT-PCR detection of orthohantavirus RNA in tissues of soricine shrews.

Species	Country	State	Year	RT-PCR Positive/Tested
*Sorex monticola*	Canada	British Columbia	2006	3/10
		Yukon Territory	2004	0/1
		Yukon Territory	2005	3/17
		Yukon Territory	2006	0/1
	USA	Alaska	1998	0/1
		Alaska	2001	3/9
		Alaska	2002	0/7
		Alaska	2005	0/6
		Colorado	1994	1/1
		New Mexico	1995	0/1
		New Mexico	1996	2/5
		New Mexico	1998	1/4
		New Mexico	2000	1/4
		Utah	1994	0/1
		Utah	1997	0/1
*Sorex palustris*	Canada	Yukon Territory	2005	1/4
	USA	New Mexico	1998	0/3
		New Mexico	2007	0/2
		Wyoming	2007	0/1
*Sorex trowbridgii*	USA	Washington	1996	2/3
		Oregon	2003	2/24
		Oregon	2004	1/8
*Sorex vagrans*	Canada	British Columbia	1996	1/9
	USA	California	1983	1/3
		New Mexico	1994	0/1
		New Mexico	1996	0/1
		Oregon	1994	0/3
		Oregon	2003	3/17
		Oregon	2004	1/5
		Oregon	2005	2/6
*Sorex bairdi*	USA	Oregon	2005	1/1
*Sorex bendirii*	USA	Oregon	2003	0/1
		Oregon	2005	0/2
*Sorex haydeni*	USA	New Mexico	1996	1/1
Total				30/164

## Supplementary Materials

The following are available online at https://www.mdpi.com/1999-4915/11/7/637/s1. Figure S1: Geographic sampling for JMSV L segment and host cytochrome *b*, Figure S2: Maximum likelihood phylogeny for JMSV M segment, Figure S3: Maximum likelihood phylogeny for JMSV S segment, Figure S4: Tanglegram for host cytochrome *b* and JMSV M segment, Figure S5: Tanglegram for *Sorex vagrans* complex on the left, and Jemez Springs S segment on the right, Figure S6: Tanglegram for Jemez Springs L segment on the left and the S segment on the right, Table S1: Oligonucleotide primers for amplification of JMSV, Table S2: GenBank accession numbers for sequences used in this study, File S1: Sequence alignments and newick format phylogenies used in this study.

## References

[1] Brooks D.R., Ferrao A.L. (2005). The historical biogeography of co-evolution: Emerging infectious diseases are evolutionary accidents waiting to happen. J. Biogeogr..

[2] Geoghegan J.L., Holmes E.C. (2017). Predicting virus emergence amid evolutionary noise. Open Biol..

[3] Nieberding C., Morand S., Libois R., Michaux J.R. (2004). A parasite reveals cryptic phylogeographic history of its host. Proc. R. Soc. B Biol. Sci..

[4] Araujo S.B.L., Braga M.P., Brooks D.R., Agosta S.J., Hoberg E.P., Von Hartenthal F.W., Boeger W.A. (2015). Undestanding host-switching by ecological fitting. PLoS ONE.

[5] Geoghegan J.L., Duchêne S., Holmes E.C. (2017). Comparative analysis estimates the relative frequencies of co-divergence and cross-species transmission within viral families. PLoS Pathog..

[6] Maes P., Adkins S., Alkhovsky S.V., Avšič-Županc T., Ballinger M.J., Bente D.A., Beer M., Bergeron É., Blair C.D., Briese T. (2019). Taxonomy of the order Bunyavirales: Second update 2018. Arch. Virol..

[7] Torres-Pérez F., Palma R.E., Hjelle B., Holmes E.C., Cook J.A. (2011). Spatial but not temporal co-divergence of a virus and its mammalian host. Mol. Ecol..

[8] Nemirov K., Henttonen H., Vaheri A., Plyusnin A. (2002). Phylogenetic evidence for host switching in the evolution of hantaviruses carried by *Apodemus* mice. Virus Res..

[9] Kang H.J., Bennett S.N., Dizney L., Sumibcay L., Arai S., Ruedas L.A., Song J.W., Yanagihara R. (2009). Host switch during evolution of a genetically distinct hantavirus in the American shrew mole (*Neurotrichus gibbsii*). Virology.

[10] Klempa B. (2018). Reassortment events in the evolution of hantaviruses. Virus Genes.

[11] Arai S., Gu S.H., Baek L.J., Tabara K., Bennett S.N., Oh H.S., Takada N., Kang H.J., Tanaka-Taya K., Morikawa S. (2012). Divergent ancestral lineages of newfound hantaviruses harbored by phylogenetically related crocidurine shrew species in Korea. Virology.

[12] Briese T., Calisher C.H., Higgs S. (2013). Viruses of the family *Bunyaviridae*: Are all available isolates reassortants?. Virology.

[13] Trifonov V., Khiabanian H., Rabadan R. (2009). Geographic dependence, surveillance, and origins of the 2009 influenza A (H1N1) virus. N. Engl. J. Med..

[14] Arai S., Bennett S.N., Sumibcay L., Cook J.A., Song J.W., Hope A., Parmenter C., Nerurkar V.R., Yates T.L., Yanagihara R. (2008). Short report: Phylogenetically distinct hantaviruses in the masked shrew (*Sorex cinereus*) and dusky shrew (*Sorex monticolus*) in the United States. Am. J. Trop. Med. Hyg..

[15] Hennings D., Hoffmann R.S. (1977). A review of the taxonomy of the *Sorex vagrans* species complex from western North America. Occas. Pap. Museum Nat. Hist. Univ. Kansas..

[16] Demboski J.R., Cook J.A. (2001). Phylogeography of the dusky shrew, *Sorex monticolus* (Insectivora, Soricidae): Insight into deep and shallow history in northwestern North America. Mol. Ecol..

[17] Hope A.G., Panter N., Cook J.A., Talbot S.L., Nagorsen D.W. (2014). Multilocus phylogeography and systematic revision of North American water shrews (genus: *Sorex*). J. Mammal..

[18] Ling J., Smura T., Tamarit D., Huitu O., Voutilainen L., Henttonen H., Vaheri A., Vapalahti O., Sironen T. (2018). Evolution and postglacial colonization of Seewis hantavirus with *Sorex araneus* in Finland. Infect. Genet. Evol..

[19] Yashina L.N., Abramov S.A., Gutorov V.V., Dupal T.A., Krivopalov A.V., Panov V.V., Danchinova G.A., Vinogradov V.V., Luchnikova E.M., Hay J. (2010). Seewis virus: Phylogeography of a shrew-borne hantavirus in Siberia, Russia. Vector-Borne Zoonotic Dis..

[20] Kang H.J., Arai S., Hope A.G., Song J.W., Cook J.A., Yanagihara R. (2009). Genetic diversity and phylogeography of Seewis virus in the Eurasian common shrew in Finland and Hungary. Virol. J..

[21] Gu S.H., Hejduk J., Markowski J., Kang H.J., Markowski M., Połatyńska M., Sikorska B., Liberski P.P., Yanagihara R. (2014). Co-circulation of soricid- and talpid-borne hantaviruses in Poland. Infect. Genet. Evol..

[22] Ling J., Sironen T., Voutilainen L., Hepojoki S., Niemimaa J., Isoviita V.M., Vaheri A., Henttonen H., Vapalahti O. (2014). Hantaviruses in Finnish soricomorphs: Evidence for two distinct hantaviruses carried by *Sorex araneus* suggesting ancient host-switch. Infect. Genet. Evol..

[23] Asikainen K., Hanninen T., Henttonen H., Niemimaa J., Laakkonen J., Andersen H.K., Bille N., Leirs H., Vaheri A., Plyusnin A. (2000). Molecular evolution of Puumala hantavirus in Fennoscandia: Phylogenetic analysis of strains from two recolonization routes, Karelia and Denmark. J. Gen. Virol..

[24] Nemirov K., Leirs H., Lundkvist A., Olsson G.E. (2010). Puumala hantavirus and *Myodes glareolus* in northern Europe: No evidence of co-divergence between genetic lineages of virus and host. J. Gen. Virol..

[25] Holmes E.C. (2004). The phylogeography of human viruses. Mol. Ecol..

[26] Dizney L.J., Ruedas L.A. (2009). Increased host species diversity and decreased prevalence of Sin Nombre virus. Emerg. Infect. Dis..

[27] GenBank. https://www.ncbi.nlm.nih.gov/genbank/.

[28] Edgar R.C. (2004). MUSCLE: Multiple sequence alignment with high accuracy and high throughput. Nucleic Acids Res..

[29] Geneious. https://www.geneious.com/.

[30] Bruen T.C., Philippe H., Bryant D. (2006). A simple and robust statistical test for detecting the presence of recombination. Genetics.

[31] Jakobsen I.B., Easteal S. (2007). A program for calculating and displaying compatibility matrices as an aid in determining reticulate evolution in molecular sequences. Bioinformatics.

[32] Maynard J.S. (1992). Analyzing the mosaic structure of genes. J. Mol. Evol..

[33] Bruen T. (2005). PhiPack: PHI test and other tests of recombination. McGill Univ. Montr. Quebec.

[34] Stamatakis A. (2014). RAxML version 8: A tool for phylogenetic analysis and post-analysis of large phylogenies. Bioinformatics.

[35] Ronquist F., Teslenko M., van der Mark P., Ayres D.L., Darling A., Höhna S., Larget B., Liu L., Suchard M.A., Huelsenbeck J.P. (2012). MrBayes 3.2: Efficient Bayesian phylogenetic inference and model choice across a large model space. Syst. Biol..

[36] Darriba D., Taboada G.L., Doallo R., Posada D. (2012). jModelTest 2: More models, new heuristics and parallel computing. Nat. Methods.

[37] (2019). R, version 3.6.0; A Language and Envionrment for Statistical Computing.

[38] Paradis E. (2010). Pegas: An R package for population genetics with an integrated–modular approach. Bioinformatics.

[39] Lessa E.P., Cook J.A., Patton J.L. (2003). Genetic footprints of demographic expansion in North America, but not Amazonia, during the Late Quaternary. Proc. Natl. Acad. Sci..

[40] Kumar S., Stecher G., Tamura K. (2016). MEGA7: Molecular Evolutionary Genetics Analysis Version 7.0 for bigger datasets. Mol. Biol. Evol..

[41] Ho S.Y.W., Duchêne S., Duchêne D. (2015). Simulating and detecting autocorrelation of molecular evolutionary rates among lineages. Mol. Ecol. Resour..

[42] Critchlow D.E., Pearl D.K., Qian C. (1996). The triples distance for rooted bifurcating phylogenetic trees. Syst. Biol..

[43] Kuhner M.K., Yamato J. (2015). Practical performance of tree comparison metrics. Syst. Biol..

[44] Avino M., Ng G.T., He Y., Renaud M.S., Jones B.R., Poon A.F.Y. (2019). Tree shape-based approaches for the comparative study of cophylogeny. Ecol. Evol..

[45] Conow C., Fielder D., Ovadia Y., Libeskind-Hadas R. (2010). Jane: A new tool for the cophylogeny reconstruction problem. Algorithms Mol. Biol..

[46] Revell L.J. (2012). phytools: An R package for phylogenetic comparative biology (and other things). Methods Ecol. Evol..

[47] Bennett S.N., Gu S.H., Kang H.J., Arai S., Yanagihara R. (2014). Reconstructing the evolutionary origins and phylogeography of hantaviruses. Trends Microbiol..

[48] Brooks D.R., Hoberg E.P., Boeger W.A., Gardner S.L., Galbreath K.E., Herczeg D., Mejía-Madrid H.H., Rácz S.E., Dursahinhan A.T. (2014). Finding them before they find us: informatics, parasites, and environments in accelerating climate change. Comp. Parasitol..

[49] Sawyer Y.E., MacDonald S.O., Lessa E.P., Cook J.A. (2019). Living on the edge: Exploring the role of coastal refugia in the Alexander Archipelago of Alaska. Ecol. Evol..

[50] Zhang Y.Z., Holmes E.C. (2014). What is the time-scale of hantavirus evolution?. Infect. Genet. Evol..

[51] Duffy S., Shackelton L.A., Holmes E.C. (2008). Rates of evolutionary change in viruses: Patterns and determinants. Nat. Rev. Genet..

[52] Raup B., Racoviteanu A., Khalsa S.J.S., Helm C., Armstrong R., Arnaud Y. (2007). The GLIMS geospatial glacier database: A new tool for studying glacier change. Glob. Planet. Change.

[53] Razzauti M., Plyusnina A., Henttonen H., Plyusnin A. (2008). Accumulation of point mutations and reassortment of genomic RNA segments are involved in the microevolution of Puumala hantavirus in a bank vole (*Myodes glareolus*) population. J. Gen. Virol..

[54] Razzauti M., Plyusnina A., Sironen T., Henttonen H., Pyusnin A. (2009). Analysis of Puumala hantavirus in a bank vole population in northern Finland: Evidence for co-circulation of two genetic lineages and frequent reassortment between strains. J. Gen. Virol..

[55] Zou Y., Hu J., Wang Z.-X.X., Wang D.-M.M., Yu C., Zhou J.-Z.Z., Fu Z.F., Zhang Y.-Z.Z. (2008). Genetic characterization of hantaviruses isolated from Guizhou, China: Evidence for spillover and reassortment in nature. J. Med. Virol..

[56] Black W.C., Doty J.B., Hughes M.T., Beaty B.J., Calisher C.H. (2009). Temporal and geographic evidence for evolution of Sin Nombre virus using molecular analyses of viral RNA from Colorado, New Mexico and Montana. Virol. J..

[57] Laenen L., Vergote V., Kafetzopoulou L.E., Wawina T.B., Vassou D., Cook J.A., Hugot J.P., Deboutte W., Kang H.J., Witkowski P.T. (2018). A novel hantavirus of the European mole, Bruges virus, is involved in frequent Nova virus coinfections. Genome Biol. Evol..

[58] Shi M., Lin X.D., Chen X., Tian J.H., Chen L.J., Li K., Wang W., Eden J.S., Shen J.J., Liu L. (2018). The evolutionary history of vertebrate RNA viruses. Nature.

[59] Souza W.M., Bello G., Amarilla A.A., Alfonso H.L., Aquino V.H., Figueiredo L.T.M. (2014). Phylogeography and evolutionary history of rodent-borne hantaviruses. Infect. Genet. Evol..

[60] Guo W.P., Lin X.D., Wang W., Tian J.H., Cong M.L., Zhang H.L., Wang M.R., Zhou R.H., Wang J.B., Li M.H. (2013). Phylogeny and origins of hantaviruses harbored by bats, insectivores, and rodents. PLoS Pathog..

[61] Castel G., Tordo N., Plyusnin A. (2017). Estimation of main diversification time-points of hantaviruses using phylogenetic analyses of complete genomes. Virus Res..

