# Supplementary material for: Complex History of Codiversification and Host Switching of a Newfound Soricid-Borne Orthohantavirus in North America
Source: Viruses. 2019 Jul 11;11(7):637. doi: 10.3390/v11070637 (PMC6669566; doi:10.3390/v11070637)
Supplement: Supplementary file 1 [file viruses-11-00637-s001.zip › viruses-529395-for conversion-supplementary/jmsv_supplementary_figures.docx]

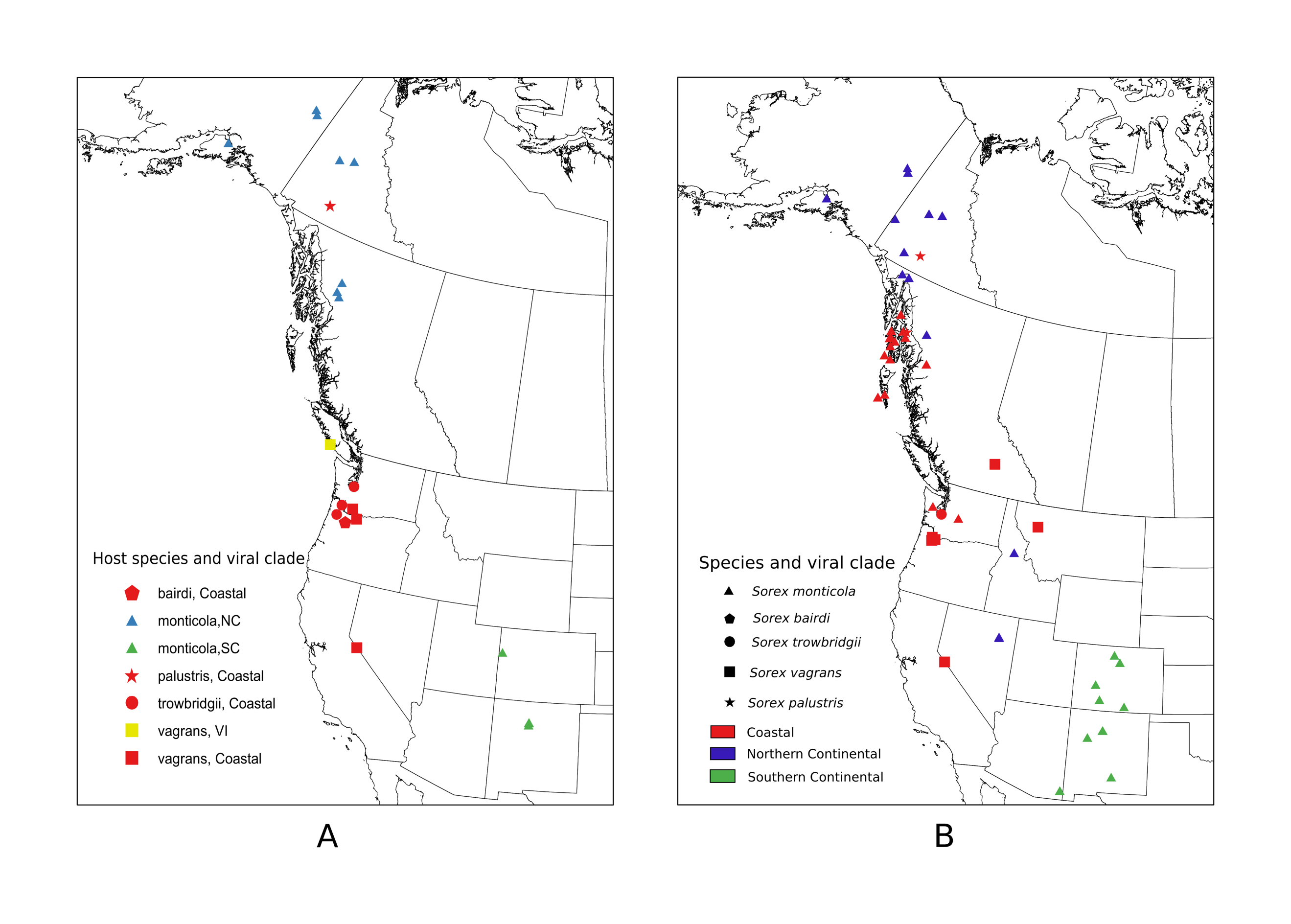


Figure S1. (**A**) Geographic distribution of Jemez Springs virus represented by sampling of the L segment. Shapes correspond to host taxa with colors representing the geographically defined clade recovered from the virus phylogeny. (**B**) Geographic sampling of the *Sorex vagrans* complex used for phylogenetic reconstruction. Host taxa is represented by different shapes with the color of the shape corresponding to geographically defined Jemez Springs virus clades.


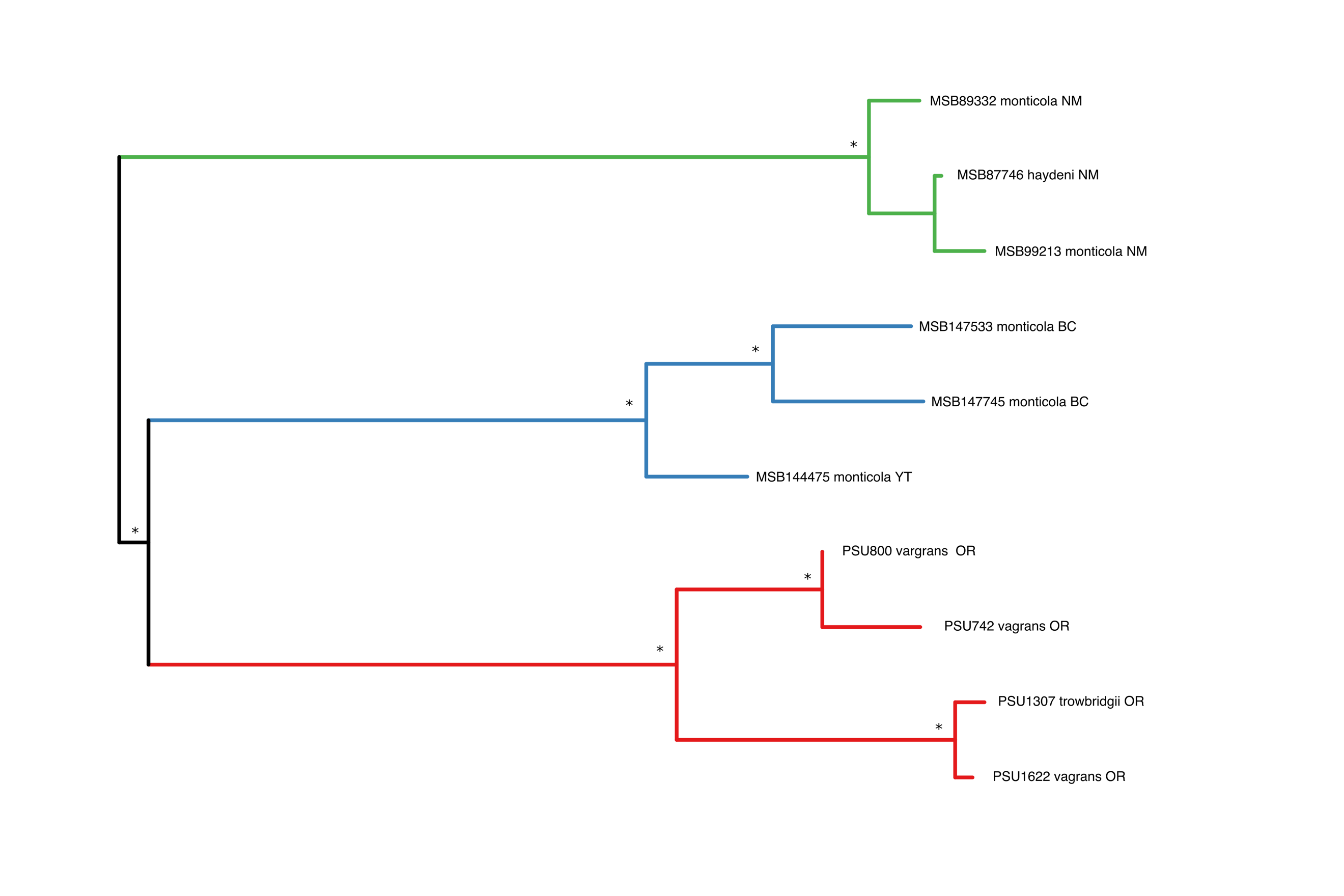


Figure S2. Maximum likelihood phylogeny of the Jemez Springs virus M segment inferred using RAxML and rooted at the midpoint. Bootstrap support values greater than 70% are indicated with an asterisk. Color-coded branches correspond to geographic clades as listed for the L segment. GenBank accession numbers for the M segment used in this study are available in Table S1.


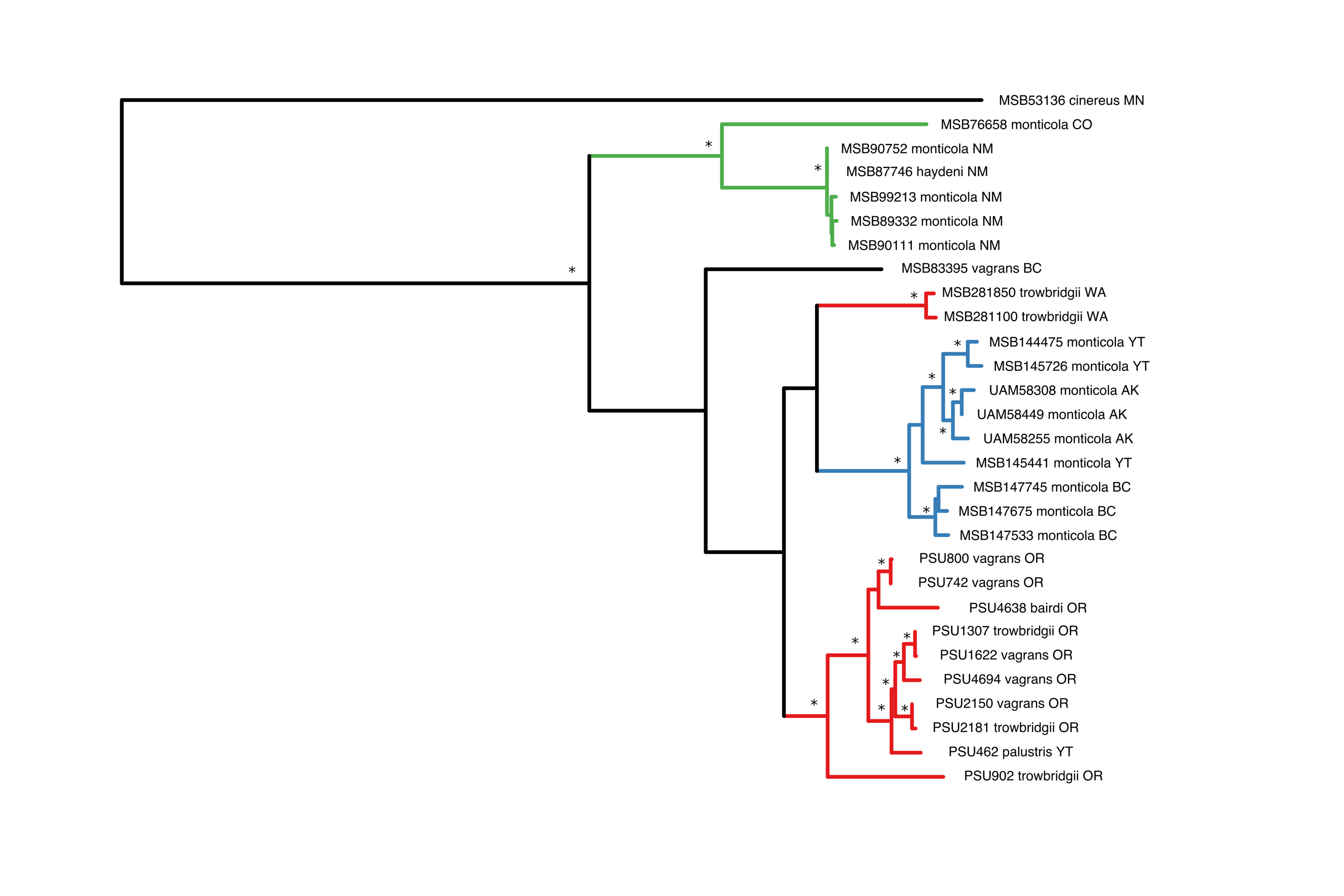


Figure S3. Maximum likelihood phylogeny of the Jemez Springs virus S segment inferred using RAxML and rooted at the midpoint. Bootstrap support values greater than 70% are indicated with an asterisk. Color-coded branches correspond to geographic clades as listed for the L segment. GenBank accession numbers for the S segment used in this study are Table S1.


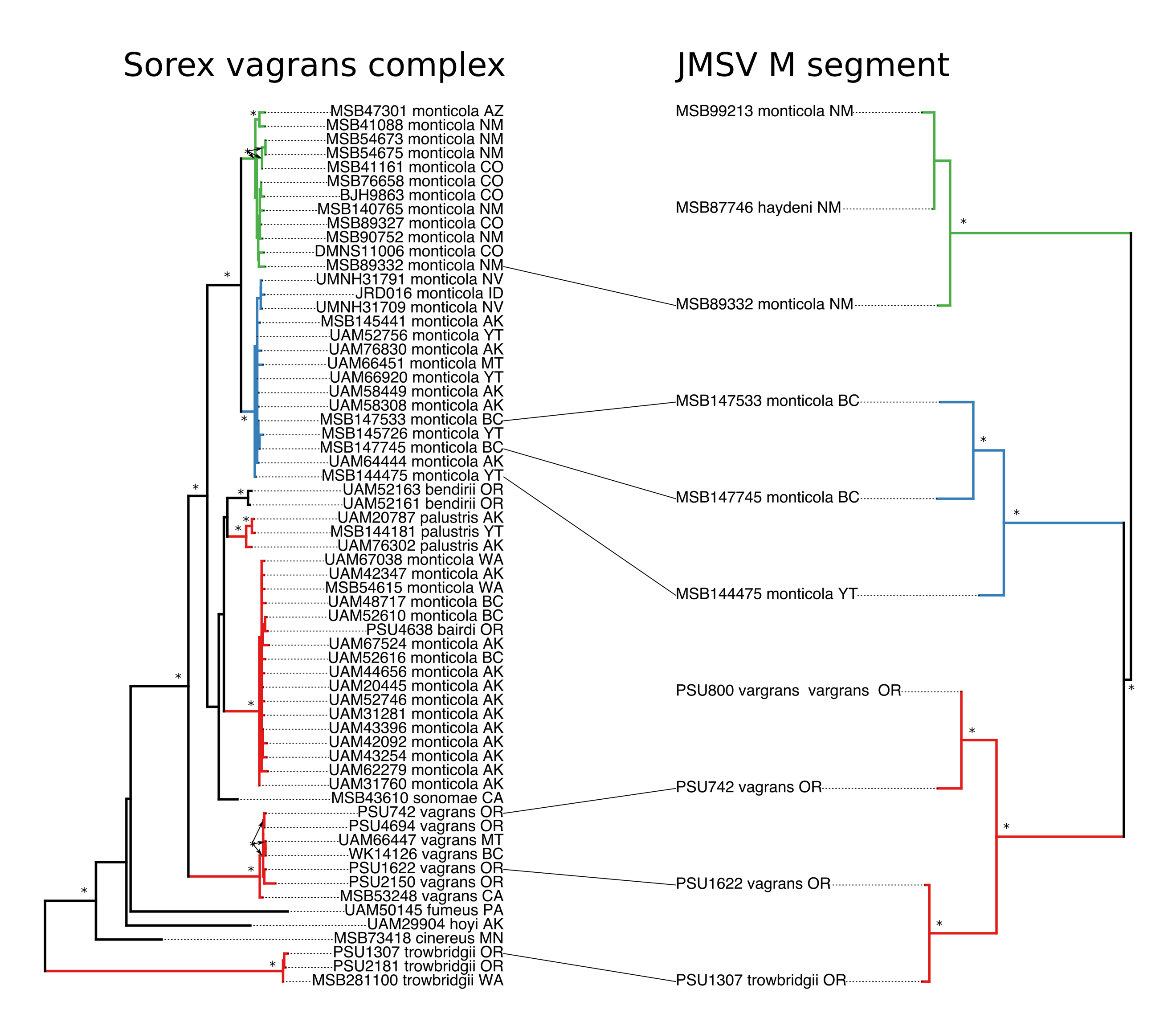


Figure S4. Tanglegram for *Sorex vagrans* complex on the left, and Jemez Springs M segment on the right. Host individuals and their associated virus isolate are illustrated with a black line. Branches are color-coded according geographically defined viral clades. Bootstrap support values greater than 70% are indicated with an asterisk.


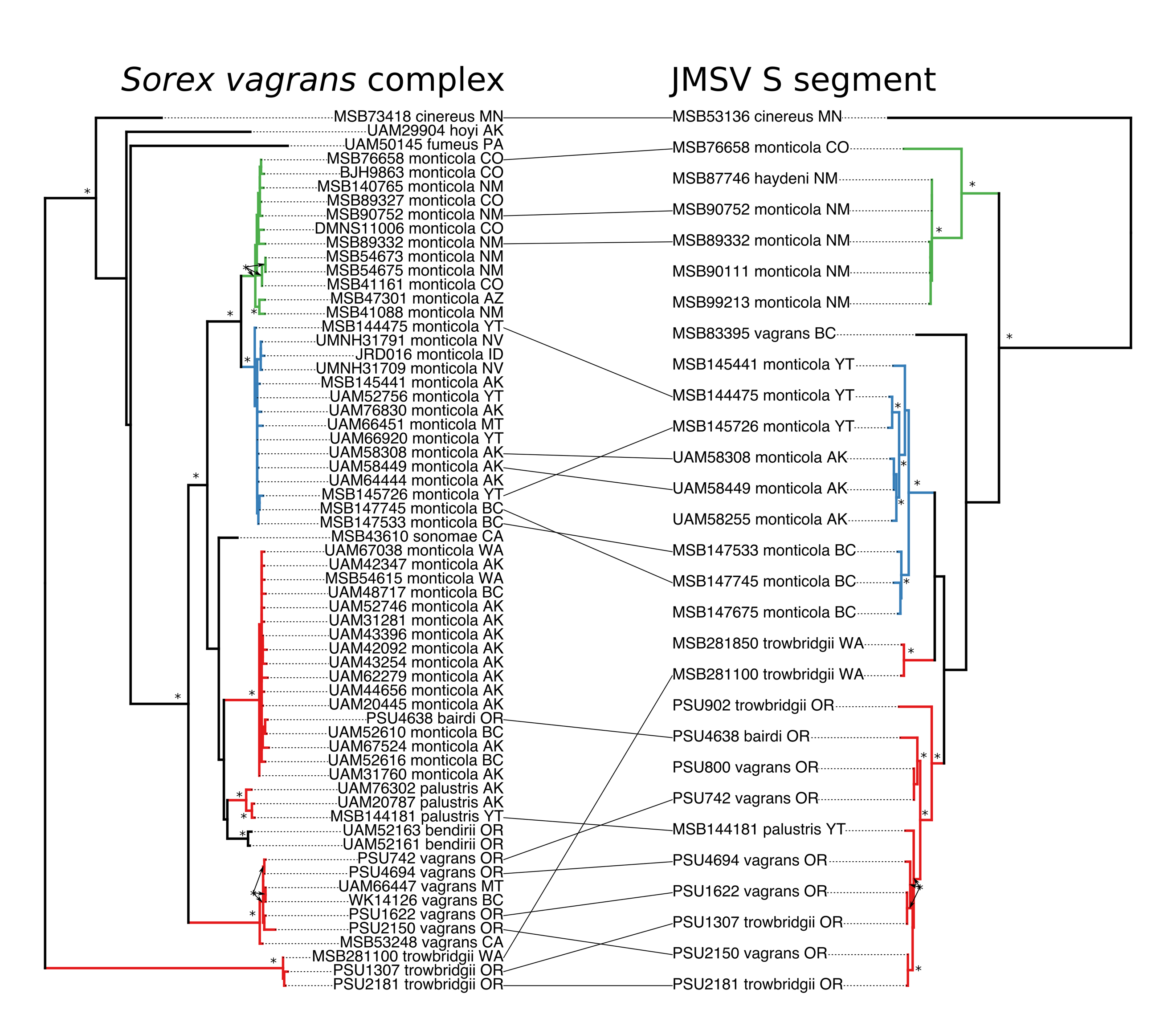


Figure S5. Tanglegram for *Sorex vagrans* complex on the left, and Jemez Springs S segment on the right. Host individuals and their associated virus isolate are illustrated with a black line. Branches are color-coded according geographically defined viral clades. Bootstrap support values greater than 70% are indicated with an asterisk.


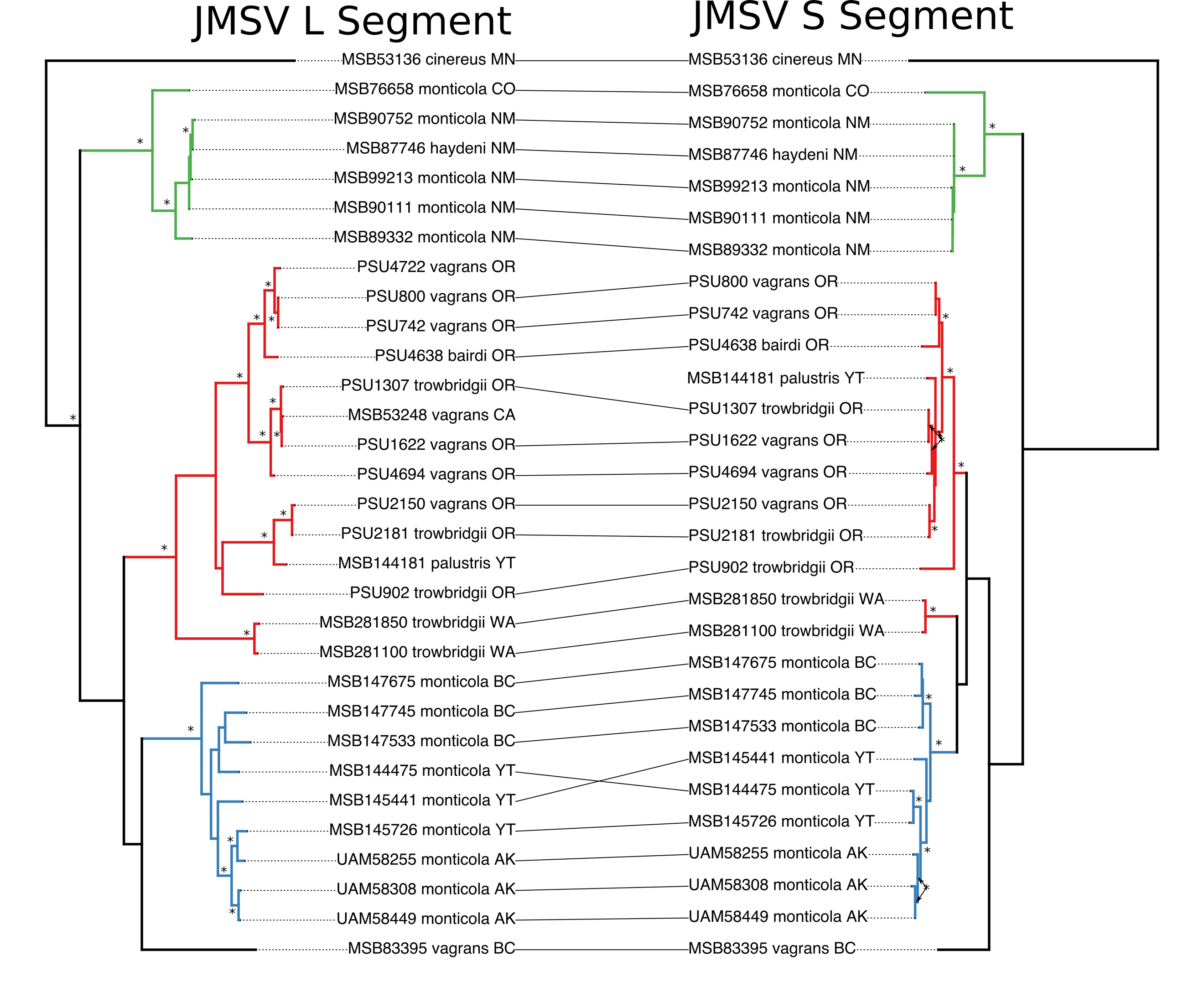


Figure S6. Tanglegram for Jemez Springs L segment on the left and the S segment on the right. Identical viruses from each segment are illustrated with a black line. Branches are color-coded according geographically defined viral clades. Bootstrap support values greater than 70% are indicated with an asterisk.

**Table S1. Oligonucleotide primers for amplification of Jemez Springs virus.**

| **Segment** | **First PCR** | **Nested PCR** |
| --- | --- | --- |
| **S** | S437F:  5'-SWGGTCARACTGCHRAYTGG-3'  Han-S1260R:  5'-CDGGRTCCATRTCATCHCCAN-3' | Han-S694F:  5'-CCNGTNATGRGNGTNATHGGNTT-3'  Cro2R:  5'-ANGAYTGRTARAANGANGAYTTYTT-3' |
| **M** | Han-M1190F:  5'-GGNCCNGGDGCWNVHTGTGA-3'  Han-M2219R:  5'-CATKCNCCATARCARTGRAA-3' | HTM-1490F:  5'-TGTGTNCCWGGNTTYCATGGNT-3'  ULU-M2178R:  5'-GYATCMANCCARTGNCCTAG-3' |
| **L** | Han-L1900F:  5'-ATGAARNTNTGTGCNATNTTTGA-3'  Han-L3000R:  5'-GCNGARTTRTCNCCNGGNGACCA-3' | Han-L2520F:  5'-ATNWGHYTDAARGGNATGTCNGG-3'  Han-L2970R:  5'-CCNGGNGACCAYTTNGTDGCATC-3' |

**Abbreviations: A, Adenine; B, C or G or T; C, Cytosine; D, A or G or T; G, Guanine; H, A or C or T; I, Inosine; K, G or T; M, A or C; N, any nucleotide; R, A or G; S, G or C; T, Thymine; V, A or C or G; W, A or T; Y, C or T.**

Table S2. GenBank accession numbers for all viral sequences and host cytochrome b used in this study.
